# Modulation of proteomic and inflammatory signals by Bradykinin in podocytes

**DOI:** 10.1016/j.jare.2020.05.021

**Published:** 2020-05-28

**Authors:** Richard Saoud, Miran A Jaffa, Aida Habib, Jingfu Zhao, Moustafa Al Hariri, Rui Zhu, Anwarul Hasan, Fuad N Ziyadeh, Firas Kobeissy, Yehia Mechref, Ayad A Jaffa

**Affiliations:** a Department of Biochemistry and Molecular Genetics, Faculty of Medicine, American University of Beirut, Lebanon; b Epidemiology and Population Health Department, Faculty of Health Sciences, American University of Beirut, Lebanon; c INSERM-U1149, Centre de Recherche sur l'Inflammation, Sorbonne Paris Cité, Laboratoire d'Excellence Inflamex, Faculté de Médecine, Site Xavier Bichat, Université de Paris, France; d Department of Chemistry and Biochemistry, Texas Tech University, Lubbock, TX 79409, United States; e Department of Mechanical and Industrial Engineering, Qatar University, Qatar; f Department of Internal Medicine, Faculty of Medicine, American University of Beirut, Lebanon

**Keywords:** Cyclooxygenase-2, ERK1/2, Liquid Chromatography Mass Spectrometry, Bradykinin 2 receptor, Podocyte proteome

## Abstract

Podocyte damage is one of the hallmarks of diabetic nephropathy leading to proteinuria and kidney damage. The underlying mechanisms of podocyte injury are not well defined. Bradykinin (BK) was shown to contribute to diabetic kidney disease. Here, we evaluated the temporal changes in proteome profile and inflammatory signals of podocytes in response to BK (10^−7^M). Protein profile was evaluated by liquid chromatography mass Spectrometry (LC-MS/MS) analysis. Proteome profile analysis of podocytes treated with BK (10^−7^M) for 3 and 6 h, revealed 61 proteins that were differentially altered compared to unstimulated control podocytes. Pathway enrichment analysis suggested inhibition of cell death pathways, engagement of cytoskeletal elements and activation of inflammatory pathways. One of the inflammatory proteins that was identified to be induced by BK treatment is Prostaglandin (PG) H Synthase-2 (Cyclooxygenase-2, COX-2). In addition, BK significantly induced the production and release of PGE_2_ and this effect was inhibited by both COX-2 and MEK Kinase inhibitors, demonstrating that the production of PGE_2_ by BK is mediated via COX-2 and MAPK-dependent mechanisms. These findings provide a global understanding of the effector modulated proteome in response to BK and also reveal BK as an important modulator of inflammation and a potential player in podocyte injury.

## Graphical abstract

**Figure:**
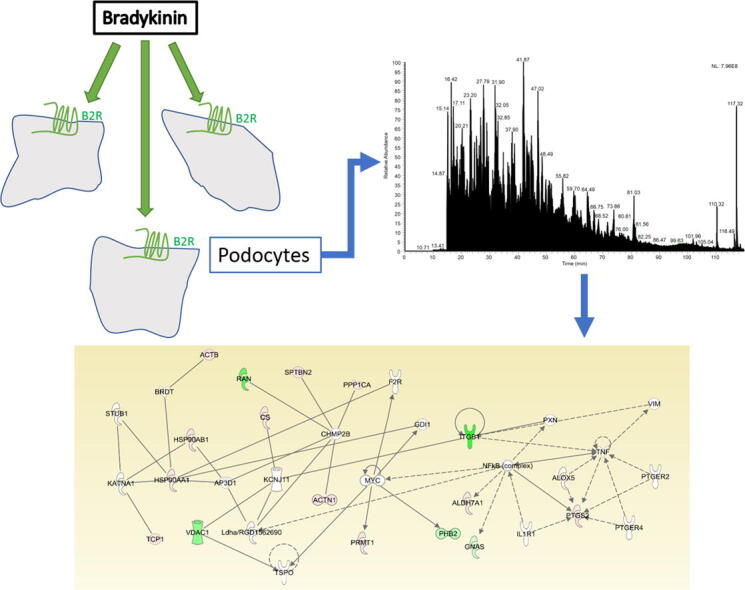

## Background

Diabetic kidney disease (DKD) is the leading cause of end-stage kidney disease [1], and it has long been recognized that the high mortality due to cardiovascular disease in diabetics with nephropathy suggested a strong association between microvascular and macrovascular pathologies in diabetes [2]. Podocyte loss has been identified as a hallmark for the progression of DKD, where the loss of podocytes has been shown to be correlated with an increase in albumin excretion rate (AER) [3], [4]. Hemodynamic injury within the glomerulus such as the dysregulation of glomerular filtration rate (GFR) has been such as a key feature contributing to development and progression of DKD [5].

Podocytes are epithelial cells lining the Bowman’s capsule in the glomerulus, as they form the third barrier of filtration preceded by the fenestrated endothelial glomerular cells and the glomerular basement membrane [6]. Podocytes possess a large number of foot processes forming slits, which filters fluid after passing through the first two barriers of filtration. The slit diaphragm serves as a signaling hub for the podocytes, with many structural proteins play their role in regulating the reactive elements of the glomerular filtration system, and thus are implicated in renal damage when dysfunctions occur with these proteins. Anatomically, it has been established that podocyte injury is a hallmark of diabetic nephropathy [3], while mesangial matrix expansion [7] and reduction in glomerular endothelial fenestration have been correlated with its progression [8]. The maladaptive factors that are accountable for these signals leading to DKD are yet not fully defined.

The Kallikrein-Kinin system has been implicated as a key player in the development of renal injury in DKD. Higher plasma prekallikrein levels were associated with higher AER in type 1 diabetic patients [9]. The AER and glomerular and tubular injury were attenuated in diabetic bradykinin 2 receptor (B_2_R^−/−^) null mice compared to wild-type diabetic B_2_R^+/+^ mice [10], [11]. In addition, previous findings from our renal global genome profiling study identified a number of dysregulated oxidative stress genes in diabetic B_2_R^−/−^ mice known to be essential to the pathogenesis of DKD [12].

It is of note that the comparative proteomics discipline is increasingly utilized to identify new biomarkers in disease and experimental states. The advent of advanced liquid chromatography/tandem mass spectrometry (LC-MS/MS) proteomics platforms has made possible the construction of the protein profile of a cell at a given time and physiological/pathological state [13]. Hays et al. detected proteins belonging to the cytoskeletal activity functional network utilizing proteomics analysis of conditionally immortalized murine podocytes [14]. In addition, utilizing a combined proteome and transcriptome analysis of podocytes revealed a number of significantly altered proteins are linked to podocyte cytoskeleton and protein transport [15]. Furthermore, in a recent study by Rinchen et al in native mouse podocytes using a combined proteomic and transcriptomic approach provided new insights into candidate markers of podocyte biology [16]. A potential role for engagement of bradykinin 2 receptor (B_2_R) in podocyte biology has not been fully explored. In the present manuscript, we use advanced proteomics data analysis to determine the protein profile of cultured rat podocytes treated with bradykinin (BK). Pathway enrichment analysis was then performed to broaden our understanding of the cellular processes and networks occurring as a result of BK treatment, and to identify key players in the signaling pathway downstream of activation of B_2_R.

## Material and methods

### Materials

Bradykinin (SC090), culture and reagents were all purchased from Sigma-Aldrich (MO, St Louis, USA). DC Protein^TM^ assay, electrophoresis reagents and Clarity^TM^ Western ECL were purchased from Bio-Rad (Hercules, California, USA). PD 98,059 and Ibuprofen were ordered from Cayman Co (MI, Ann Arbor, USA). Antibodies used as follow: Anti-β-Actin (Abcam, Cambridge, UK, ab8227), Phospho-p44/42 MAPK (ERK1/2) rabbit mAb (Cell Signaling Technology, Danvers, MA, USA, 4370 s), p42/44 MAPK rabbit mAb (ERK1/2) (Cell Signaling Technology, 4695 s), Peroxidase-conjugated affinipure donkey anti-rabbit IgG (Jackson ImmunoResearch, West Grove, PA, USA) 711-035-152), or affinipure donkey anti-mouse IgG (Jackson Immune, 715–035-150), mouse monoclonal antibody anti-COX-2 (clone COX214). HPLC water was purchased from Avantor (Valley Center, PA, USA), acetonitrile (ACN) from Thermo Fisher Scientific (Waltham, MA, USA), trypsin/Lys-C mixture was produced by Promega (Madison, WI, United States) and formic acid (FA) was obtained from (TCI America, Portland, OR, USA).

### Mass spectrometry

#### Extraction and tryptic digestion of proteins

Conditionally immortalized rat podocytes grown in RPMI 1640 culture media containing insulin, PS, Glutamine, fetal bovine serum (FBS) and HEPES were used in our studies. The starvation media was deprived of FBS and Insulin. Serum starved cells were treated with BK (10^−7^M) for 3 and 6 h. The concentration of BK was selected based on our prior studies assessing the effects of various doses of BK on renal and vascular cells. With respect to the two time points (3 and 6 h), we were interested in determining the temporal changes in early response differentially expressed proteins in podocytes as a result of BK stimulation. Beads beating method was next employed for cell lysis and protein extraction. Briefly, zirconium beads (diameter 0.4 mm) and a 200 μL aliquot of aqueous solution containing 5% sodium deoxycholate (SDC) were added to the cell samples. The mixture was homogenized using Beadbug microtube homogenizer at 4 °C at 4000 rpm. Six times of 30-second-homogenization plus another 30-second-pause were performed to break the cells. The cell lysate was sonicated for 30 min at 0 °C to enhance the extraction of proteins. After centrifuging at 14,800 g for ten minutes, the supernatant was diluted ten X with 50 mM ammonium bicarbonate buffer. BCA protein assay kit (Thermo Scientific; Rockford, IL) was used for Protein determination. To reduce the protein sample, 1.5 μL of DTT (200 mM) was added for 45 min at 60 °C. 6 μL of Iodoacetamide (200 mM) to alkylate the sample was added in the dark for 45 min at 37.5 °C. Tryptic digestion was conducted by incubating with trypsin (0.4 µg) 37 °C for 18 h and microwave-digested for 30 min at 45 °C. To quench the digestion, 0.5 μL of formic acid (FA) was added. Meanwhile, SDC detergent was precipitated in the acidic condition. Samples were then mixed thoroughly and centrifuged 10 min at a speed of 14,800g. Digested peptides (Supernatant) were dried and mixed with 0.1% formic acid and 2% acetonitrile (ACN) for LC-MS/MS analysis.

#### LC-MS/MS assay

Data from LC-MS/MS was collected utilizing the Dionex Ultimate 3000 nano-LC system (Thermo Fisher Scientific) interfaced to an LTQ Orbitrap Velos mass spectrometer with a nano-ESI source (Thermo Fisher Scientific). A 1 µg aliquot of tryptic digests was analyzed by the LC-MS/MS. Generated peptides were purified utilizing the C18 PepMap 100 trap column, 3 µL/min flow rate. Peptide separation was done with a C18 Acclaim PepMap RSLC column (75 µm I.D. × 15 cm, 2 µm particle sizes, 100 Å pore sizes) with a flow rate of 350 nl/min. Mobile phase comprised of solution A (2% ACN/0.1% FA in HPLC water) and solution B (99.9% ACN/0.1% FA). The separation method was used according to our previously published protocol [13]. The MS was operated in positive ion mode. Two scan events at data dependent acquisition mode were employed in the MS/MS analysis. The detailed methodology for the scan events were used according to our previously published methods [13].

#### LC-MS/MS data analysis

The data from Liquid chromatography-electrospray ionization-tandem mass spectrometry LC-ESI-MS/MS was utilized to produce mascot generic format file (*.mgf) employing the Proteome Discover version 1.2 software (Thermo Scientific). Data were examined utilizing the SwissProt database (Rattus) in MASCOT version 2.4 (Matrix Science Inc., MA, USA). MASCOT identified peptides and proteins were reduced by PeptideProphet and ProteinProphet algorithms. Peptide identifications with probabilities higher than 95% were accepted, whereas those with probabilities higher than 99% containing a minimum of 2 peptides were allowed.

#### Pathway enrichment analysis

Pathway Studio software (version 11. Elsevier) along with Ingenuity Pathway Analysis software (IPA, Qiagen, Ingenuity® Systems) were used to assess functional correlations among the different treatment cohorts to assess associations, relationships, biological processes and molecular functions within the different treatment groups. For the statistical analysis, Fisher’s test was employed to evaluate nonrandom associations (protein–protein interaction and pathways). Gene symbols were imported for each experimental data set and its corresponding log2 fold change expression values were uploaded for functional analysis using IPA and pathway studio knowledgebase [13].

#### Cell treatment and analysis

Quiescent podocytes cultured in 6-well plates were treated with BK (10^−7^ M) in the presence and absence of MEK (MAP kinase kinase) inhibitor (PD-98059, 25x10^−6^ M) and/or a non-selective COX-1/2 inhibitor, Ibuprofen (10^−6^ M) for various time points. PGE_2_ levels were measured in the supernatants by Enzyme-immunoassay (EIA) as described [17]. Podocytes were lysed in 200 μL of lysis buffer (50 mM Tris, pH 8, 150 mM NaCl, 0.1% SDS, 0.5% deoxycholic acid, 1% NP40, plus protease and phosphatase inhibitors leupeptin 2 μg/ml, aprotinin 2 μg/ml, sodium orthovanadate 2 mM, PMSF 1 mM, Benzamidine 1 mM, sodium pyrophosphate 1 mM, and sodium fluoride 10 mM). Concentration of protein was assessed using the Lowry assay with BSA as standard. For western blotting of COX-2 levels and phosphorylation of ERK1/2, 20–30 μg of total proteins were separated by SDS-PAGE (polyacrylamide gel electrophoresis) as previously described [18], [19]. Protein bands were revealed by ECL kit on the Bio-Rad ChemiDoc MP imaging System. Protein bands were quantified using ImageJ software (NIH, USA), and a ratio to actin or total ERK1/2 was determined and expressed relative to untreated cells as control.

### Statistical analysis

Simple descriptive statistics were initially conducted on the mean for each group and for each time point. The normality of the data was determined graphically using the Q-Q and numerically utilizing Shapiro-Wilk test for normality. When normality was met, independent *t*-test was conducted to compare the means between the groups and p-values were reported based on the test of homogeneity. When the normality of the data could not be assumed the alternative nonparametric Mann-Whitney *U* test was conducted as an alternative approach. Data were expressed as mean ± SE and significance was considered at p < 0.05.

***Availability of Proteomic data***. The proteomics data generated from LC-MS/MS was deposited in the PRIDE Archive (http://www.ebi.ac.uk/pride/archive/) via the PRIDE partner repository with the data set identifier PXD010015. Username: reviewer26015@ebi.ac.uk Password: drhw12lo.

## Results

### Proteomic analysis

**Fig. 1:**
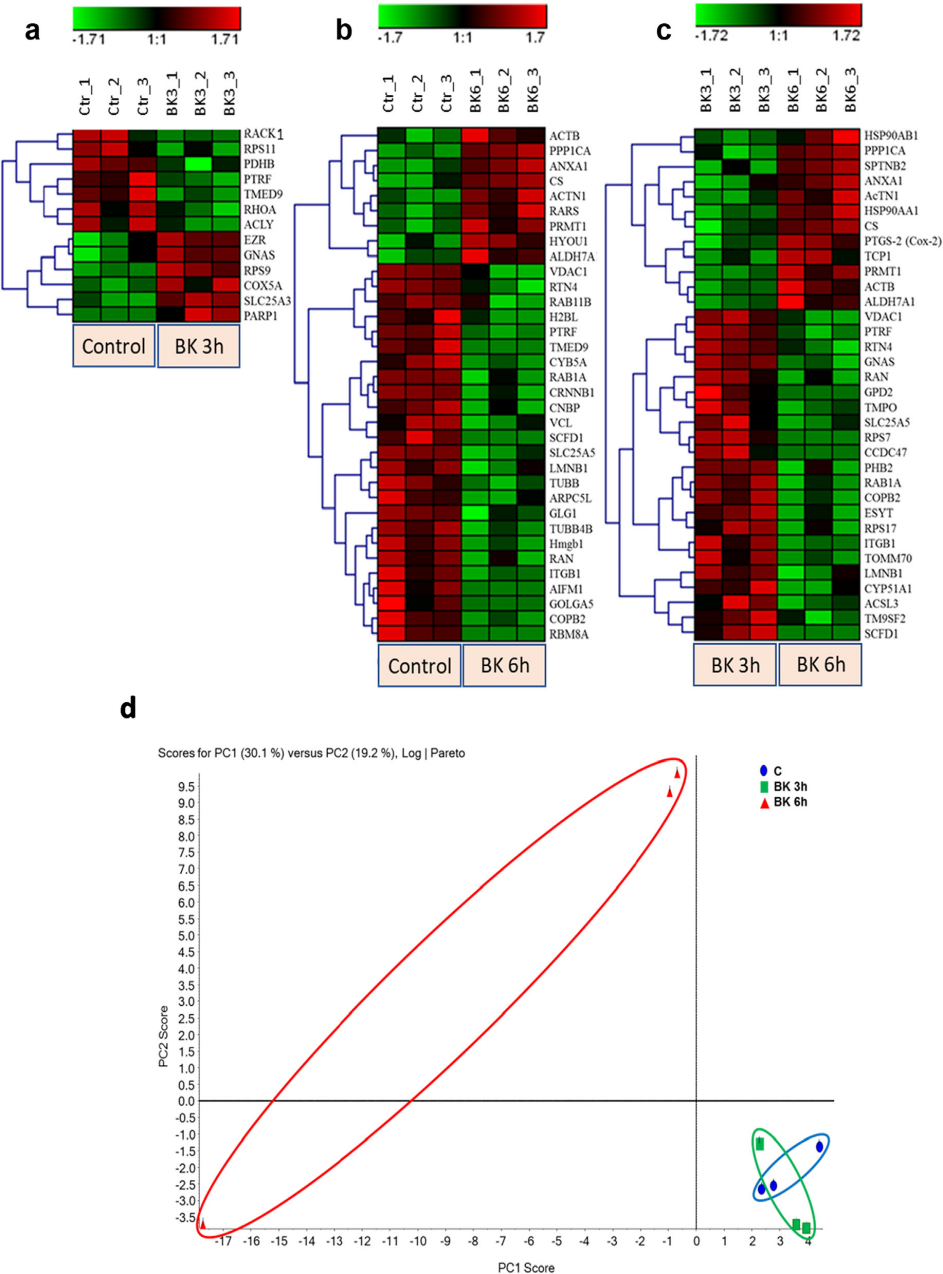
Hierarchical clustering (heat maps) and X showing the different proteins expression profile in the 3 experimental groups: (a) Control vs 3 h BK, (b) Control vs 6 h BK, and (c) 3 h vs 6 h BK. Each row represents a different protein while each column represents one triplicate from the corresponding experimental group. Green color represents downregulation of protein expression, whereas the red color represents upregulation of protein expression. Color intensity reflects the expression level of the proteins (scale). The label on the right-hand side of the heat maps represents the accession number of the proteins. (d) Principal Component Analysis (PCA) of BK-stimulated podocytes. The total normalized expression data of the proteins was used to depict the scatter plots of the first (X) and the second (Y) principal components. The numbering of the nodes is an identification of the samples. Letters used in the labels of the nodes represent the repeats. Blue oval encircles the control samples, green oval encircles the BK 3 h stimulated samples, and red oval encircles the BK 6 h stimulated samples. (For interpretation of the references to color in this figure legend, the reader is referred to the web version of this article.)

Proteome profiling of podocyte cell protein abundance was done using LC-MS/MS followed by MASCOT analysis of the resulting protein spectra from control and BK stimulated cells. Analysis showed a regulation of 280 proteins, reduced to 61 proteins when adjusting for significance (p < 0.05). These hits were split among 3 groups comparing the change in protein profile between each defined experimental condition, resulting in the analytical groups, control vs 3 h BK treatment (Fig. 1a), control vs 6 h BK treatment (Fig. 1b), and 3 h vs 6 h BK treatment (Fig. 1c). The pattern of significant protein upregulation and downregulation in the 3 analytical groups is shown in Table 1. A full list of the significantly modified proteins by BK (p < 0.05) along with their accession numbers, protein symbols, fold change, and log2 fold change are presented in Table 1, Table 2, Table 3. The hierarchal clustering of the expressed protein profile in control and BK treated cells are depicted by the heat maps shown in Fig. 1, where a row represents a protein and a column represents one of the triplicates for each experimental group. Some observed trends in the different heat maps is an increase in regulated proteins past the 3-hour BK treatment point, and the overlapping of a number of the modified proteins between the “Control vs BK 6 h” and “BK 3 h vs BK 6 h” groups. A number of proteins that are of particular interest are dysregulated by BK treatment including PTGS-2 (COX-2), RhoA, β-Catenin and Integrin-β1.

**Table 1:** Comparative list of proteins in the “Control vs 3 h BK treatment” experimental group.

Accession	Protein Name	Symbol	p-value	Fold change	Log2 Fold change
P16638	ATP Citrate Lyase	ACLY	0.049	0.149	−2.748
P11240	Cytochrome *c* oxidase subunit 5A	COX5A	0.031	5.344	2.418
P31977	Ezrin	EZR	0.033	1.358	0.442
P63095	GNAS Complex locus	GNAS	0.034	1.318	0.399
P85125	Polymerase I and transcript release factor release factor	PTRF	0.021	0.662	−0.596
P49432	Pyruvate dehydrogenase	PDHB	0.037	0.376	−1.409
P61589	Ras homolog family member A	RHOA	0.047	0.507	−0.981
P63245	Receptor for activated C Kinase1	RACK1	0.044	0.306	−1.707
P62282	Ribosomal Protein S11	RPS11	0.042	0.186	−2.424
P29314	Ribosomal Protein S9	RPS9	0.002	1.844	0.883
P16036	Solute carrier family 25	SLC25A3	0.003	2.562	1.357
Q5I0E7	Transmembrane p24 trafficking protein 9	TMED9	0.014	0.104	−3.262

**Table 2:** Comparative list of proteins in the “Control vs 6 h BK treatment” experimental group.

Accession	Protein Name	Symbol	p-value	Fold change	Log2 Fold change
P60711	Actin Beta	ACTB	0.043	2.159	1.111
Q9Z1P2	Actinin alpha 1	ACTN1	0.008	1.959	0.970
Q64057	Aldehyde Dehydrogenase 7 family member A1	ALDH7A1	0.040	3.771	1.915
P07150	Annexin A1	ANXA1	0.004	2.013	1.009
Q9JM53	Apoptosis inducing factor, mitochondrial	AIFM1	0.016	0.001	−10.751
P40329	arginyl-tRNA synthetase	RARS	0.015	2.237	1.161
A1L108	Actin-related protein 2/3 complex subunit 5-like protein	ARPC5L	0.042	0.186	−2.425
Q9WU82	Catenin Beta 1	CTNNB1	0.009	0.171	−2.545
P62634	CCHC-type zinc finger nucleic acid binding protein	CNBP	0.024	0.198	−2.339
Q8VHF5	Citrate Synthase	CS	0.009	5.960	2.575
O35142	Coatomer protein complex subunit beta 2	COPB2	0.017	0.101	−3.308
P00173	Cytochrome b5 Type A	CYB5A	0.008	0.082	−3.609
Q62638	Golgi glycoprotein 1	GLG1	0.026	0.346	−1.531
Q3ZU82	Golgin 5A	GOLGA5	0.042	0.001	−10.961
P63159	High mobility group box 1	Hmgb1	0.009	0.112	−3.159
Q00715	Histone Cluster 1, H2bl	–	0.039	0.721	−0.471
Q63617	Hypoxia up-regulated 1	HYOU1	0.027	1.539	0.622
P49134	Integrin subunit Beta 1	ITGB1	0.014	0.152	−2.713
P70615	Lamin B1	LMNB1	0.038	0.312	−1.681
P85125	Polymerase I and transcript release factor release factor	PTRF	0.002	0.297	−1.750
Q63009	Protein arginine methyltransferase 1	PRMT1	0.020	4.450	2.154
P62138	Protein Phosphatase 1	PPP1CA	0.001	3.394	1.763
O35509	RAB11B, member RAS oncogene family	RAB11B	0.046	0.454	−1.138
Q6NYB7	RAB1A, member RAS oncogene family	RAB1A	0.015	0.314	−1.670
P62828	RAN, member RAS oncogene family	RAN	0.045	0.224	−2.161
Q9JK11	Reticulon 4	RTN4	0.018	0.497	−1.007
Q27W01	RNA binding motif protein 8A	RBM8A	0.008	0.001	−10.174
Q62991	Sec 1 family domain	SCFD1	0.006	0.000	−11.026
Q09073	Solute carrier family 25	SLC25A5	0.003	0.608	−0.717
Q5I0E7	Transmembrane p24 trafficking protein 9	TMED9	0.006	0.000	−11.362
Q6P9T8	Tubulin Beta 4B class Ivb	TUBB4B	0.007	0.783	−0.353
P69897	Tubulin Beta class I	TUBB	0.020	0.895	−0.160
P85972	Vinculin	VCL	0.028	0.242	−2.049
Q9Z2L0	Voltage dependent anion channel 1	VDAC1	0.019	0.409	−1.289

**Table 3:** Comparative list of proteins in the “3hl vs 6 h BK treatment” experimental group.

Accession	Protein Name	Symbol	p-value	Fold change	Log2 Fold change
P60711	Actin Beta	ACTB	0.020	2.608	1.383
Q9Z1P2	Actinin alpha 1	ACTN1	0.014	1.698	0.764
Q63151	acyl-CoA synthetase long-chain family member 3	ACSL3	0.044	0.246	−2.025
Q64057	Aldehyde Dehydrogenase 7 family member A1	ALDH7A1	0.035	3.099	1.632
P07150	Annexin A1	ANXA1	0.027	1.572	0.652
Q8VHF5	Citrate Synthase	CS	0.020	2.966	1.569
O35142	Coatomer protein complex subunit beta 2	COPB2	0.008	0.139	−2.851
Q5U2X6	Coiled-coil domain containing 47	CCDC47	0.029	0.000	−11.177
Q64654	Cytochrome P450 family 51 subfamily A member 1	CYP51A1	0.045	0.203	−2.297
Q9Z1X1	Extended synaptotagmin 1	ESYT1	0.016	0.177	−2.494
P35571	Glycerol-3-phosphate dehydrogenase 2	GPD2	0.041	0.000	−11.906
P63095	GNAS Complex locus	GNAS	0.004	0.615	−0.700
P82995	hsp90 alpha family class A member 1	HSP90AA1	0.004	2.293	1.197
P34058	hsp90 alpha family class B member 1	HSP90AB1	0.044	2.382	1.252
P49134	Integrin subunit Beta 1	ITGB1	0.009	0.120	−3.055
P70615	Lamin B1	LMNB1	0.042	0.325	−1.623
P85125	Polymerase I and transcript release factor release factor	PTRF	0.007	0.449	−1.154
Q5XIH7	Prohibitin 2	PHB2	0.035	0.494	−1.018
P35355	Prostaglandin-endoperoxide synthase 2	PTGS2	0.016	1.666	0.737
Q63009	Protein arginine methyltransferase 1	PRMT1	0.007	15.617	3.965
P62138	Protein Phosphatase 1	PPP1CA	0.015	2.926	1.549
Q6NYB7	RAB1A, member RAS oncogene family	RAB1A	0.011	0.268	−1.899
P62828	RAN, member RAS oncogene family	RAN	0.038	0.198	−2.334
Q9JK11	Reticulon 4	RTN4	0.018	0.473	−1.080
P04644	Ribosomal protein S17	RPS17	0.047	0.219	−2.191
P62083	Ribosomal protein S7	RPS7	0.006	0.000	−12.192
Q62991	Sec 1 family domain	SCFD1	0.008	0.001	−10.543
Q09073	Solute carrier family 25	SLC25A5	0.048	0.621	−0.687
Q9QWN8	Spectrin Beta, non-erythrocytic 2	SPTBN2	0.018	2.452	1.294
P28480	T-complex 1	TCP1	0.047	6.174	2.626
Q62733	Thymopoietin	TMPO	0.039	0.223	−2.167
Q75Q39	Translocase of outer mitochondrial membrane 70	TOMM70	0.020	0.108	−3.217
Q66HG5	Trans-membrane 9 superfamily member 2	TM9SF2	0.044	0.605	−0.726
Q9Z2L0	Voltage dependent anion channel 1	VDAC1	0.013	0.317	−1.656

***Principal component analysis (PCA)*:** To appreciate the global proteome variations in response to BK stimulation between the different groups, we conducted PCA, a multivariate data analysis approach employed to scheme and convert the global alterations in big datasets into two-dimensional plots to illustrate the similarities in the variations amid the diverse groups. PCA plots for BK treated groups 3 and 6 h, displayed a well-organized parting among the various groups, suggesting that the BK time-point stimulations produced unique proteomic profiles. In addition, we observed similarities or overlay amongst the PCA plot of the 3 h stimulation by BK and control groups (Fig. 1d).

### Pathway and network analysis of proteomic profiles

**Fig. 2:**
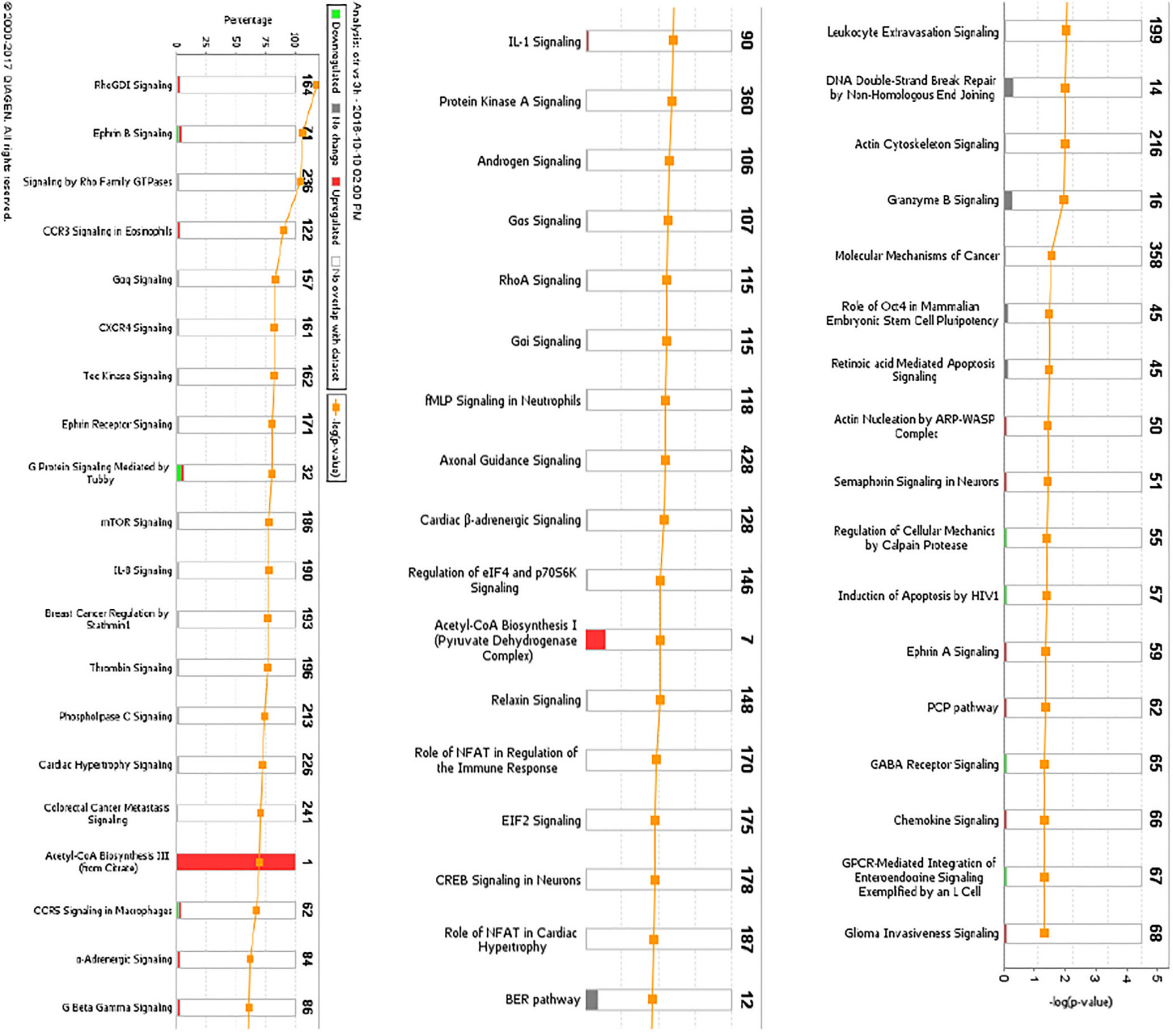

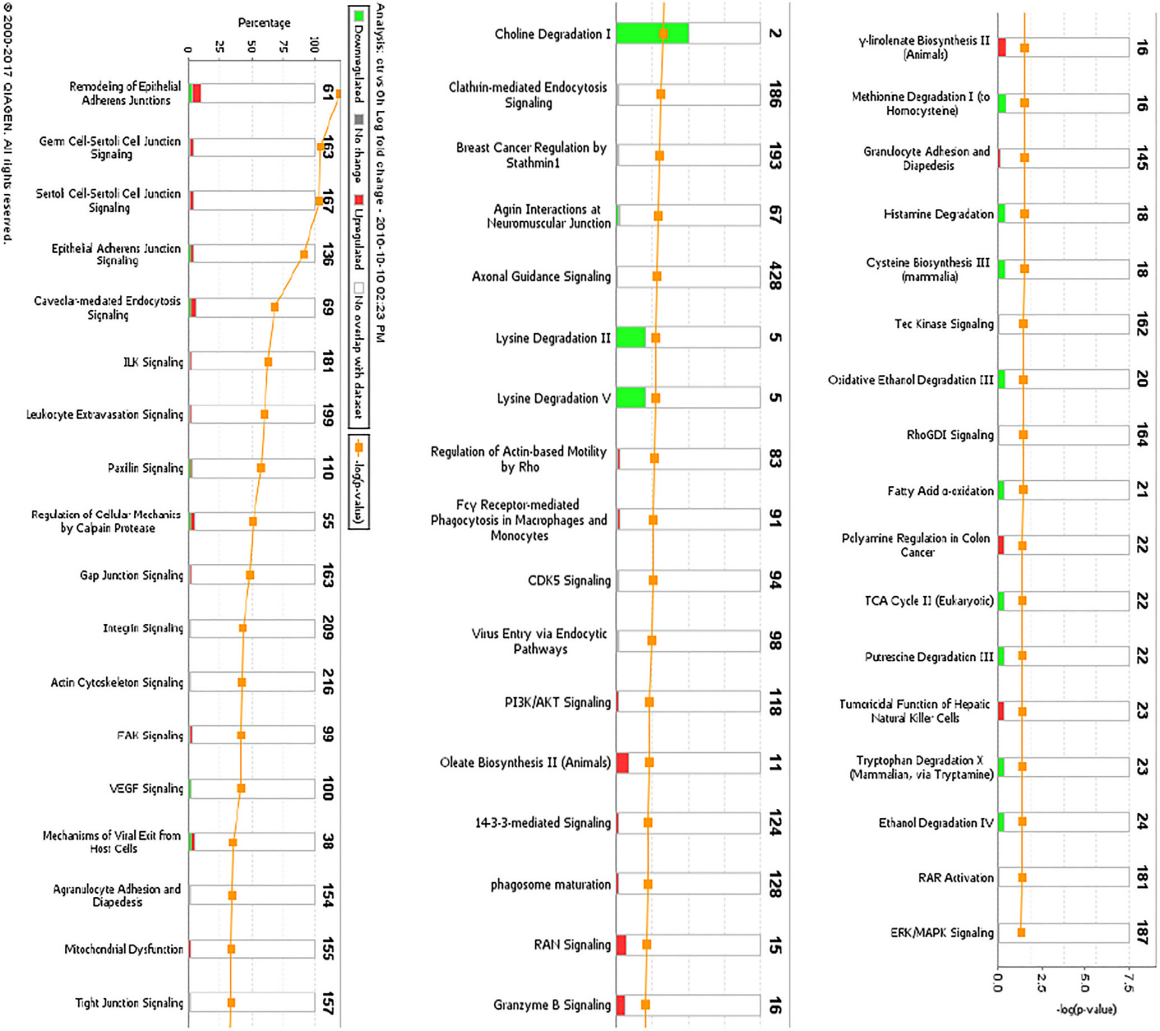

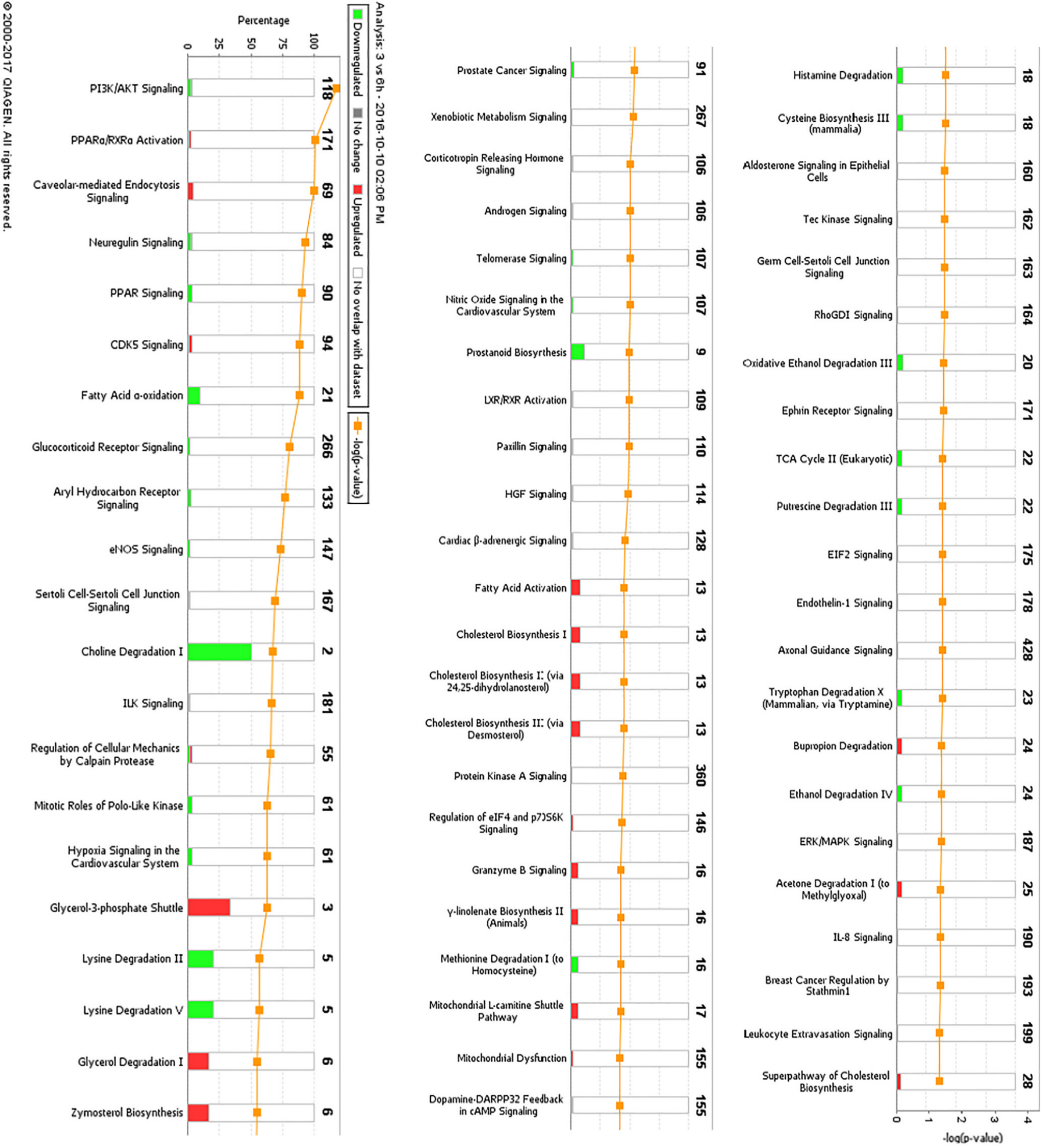
Canonical Pathway Analysis of the comparative groups in unstimulated and BK-stimulated podocytes depicting the suggested cellular mechanisms involved. **(a)** Top canonical pathways related to proteins altered in the Control vs 3 h BK. **(b)** Top canonical pathways related to proteins altered in the Control vs 6 h BK**. (c)** Top canonical pathways related to proteins altered in the 3 h vs 6 h BK. Bars show the total number of proteins identified in each pathway. The green color represents the downregulated proteins, and the red color represents the upregulated proteins. (For interpretation of the references to color in this figure legend, the reader is referred to the web version of this article.)

**Fig. 3:**
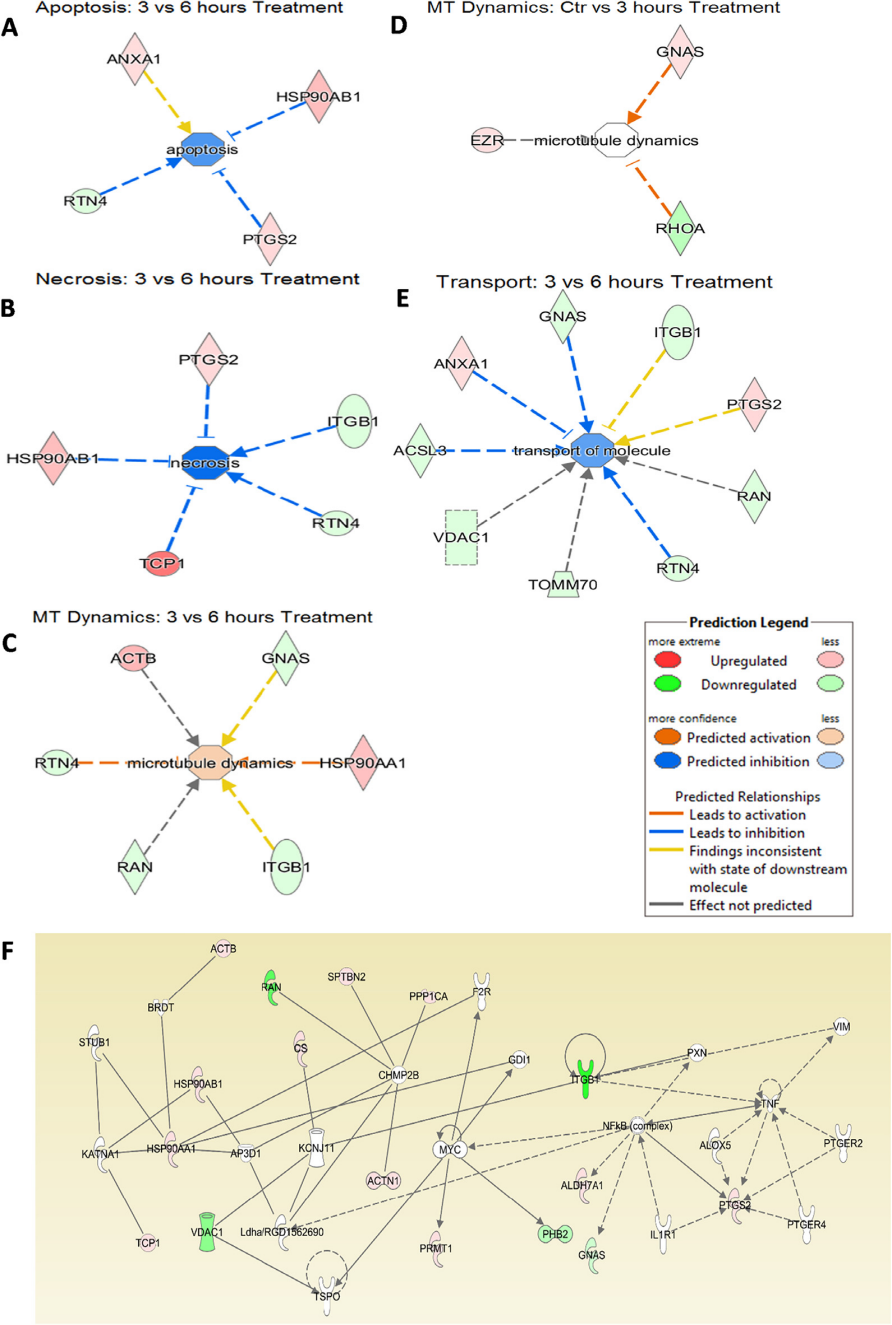
IPA’s individual pathway analysis of different proteins from the different experimental groups, linking them to a suggested promotion or inhibition of a cellular event under the umbrella of inhibition of cell death or change in cytoskeletal activity. **(a)** Inhibition of Apoptosis comparing BK 6 h treated podocytes versus controls (ANXA1: Annexin A1, HSP90AB1: Hsp90 alpha family class B member 1, RTN4: Reticulon 4, PTGS-2: Prostaglandin H-Synthase 2). **(b)** Inhibition of Necrosis comparing BK-6h treated podocytes versus controls (ITGB1: Integrin subunit Beta 1, TCP-1: T-complex 1). **(c)** Activation of Microtubule Dynamics activity comparing BK- 3h treated podocytes vs BK-6h treated podocytes (ACTB: Actin Beta, GNAS: GNAS complex locus, HSP90AA1: Hsp90 alpha family class A member 1, RAN: RAN, member RAS family oncogene). (**d)** Suggestion of Microtubule Dynamics activity comparing BK-3h treated podocytes vs control (EZR: Ezrin, RHOA: Ras homolog family member A). **(e)** Inhibition of Transport of Molecules comparing BK-3h vs. Bk-6h treated podocytes (ACSL3: acyl-CoA synthetase long-chain family member 3, TOMM70: Translocase of outer mitochondrial membrane 70, VDAC1: voltage dependent anion channel 1. **(f)** The network titled “Cell Death and Survival, Cancer, GI Disease” of the BK -3h vs. BK-6h groups in the network analysis function of IPA showing PTGS-2/COX-2 with other proteins, expressed (colored) or suggested (uncolored). PTGS-2/COX-2 is directly in contact with the proteins ALOX5 (arachidonate-5-lipoxygenase), IL1R1 (interleukin 1 receptor type 1), PTGER2 (prostaglandin E receptor 2), PTGER4 (prostaglandin E receptor4), NFκB (complex) (nuclear factor kappa-light chain-enhancer of activated B cells), TNF (tumor necrosis factor).

The differentially expressed proteome profiles in control and BK stimulated podocytes were analyzed by IPA. Fig. 2a–c depicts a graphical assessment of the canonical pathways of differentially (up- and down-regulated) proteins within and between each group. The canonical pathway analysis points to many intriguing processes that are suggested to be occurring with podocyte injury and apoptosis that includes upregulation of inflammatory cytokines and chemokines, Calpain signaling, Granzyme signaling and Ephrin B signaling. In addition, the PI3K pathway, PPAR signaling pathways, RhoA signaling, phospholipase C signaling, actin cytoskeleton signaling, and actin nucleation by ARP-WASP, remodeling of the epithelial adherens junctions, tight junction signaling, regulation by Rho of actin-based motility, ERK/MAPK signaling, NOS-III signaling, and hypoxia signaling in the cardiovascular system were shown to be among the top enriched pathways (Fig. 2a–c). Enrichment analysis of expressed proteins in podocytes stimulated with BK compared to control podocytes depicted that the most highly suspected diseases and biological functions to be involved fall under the umbrella of inhibition of cell death (inhibition of necrosis, cell death and apoptosis) and change in cytoskeletal elements (inhibition of transport of molecules and promotion of microtubule dynamics). Fig. 3a–d show the individual pathways from IPA’s “Diseases and Biological Functions” of the altered proteins. In addition network analysis depicted in Fig. 3f, showed that the upregulation of COX-2 is linked to the suggested expression/activity of the PGE receptors 2/4 (PTGER2 and PTGER4), the interleukin 1 receptor type 1 (IL1R1), tumor necrosis factor (TNF), NFκB, and arachidonate 5-Lipoxygenase (ALOX5).

### Effect of BK on COX-2 expression in podocytes

**Fig. 4:**
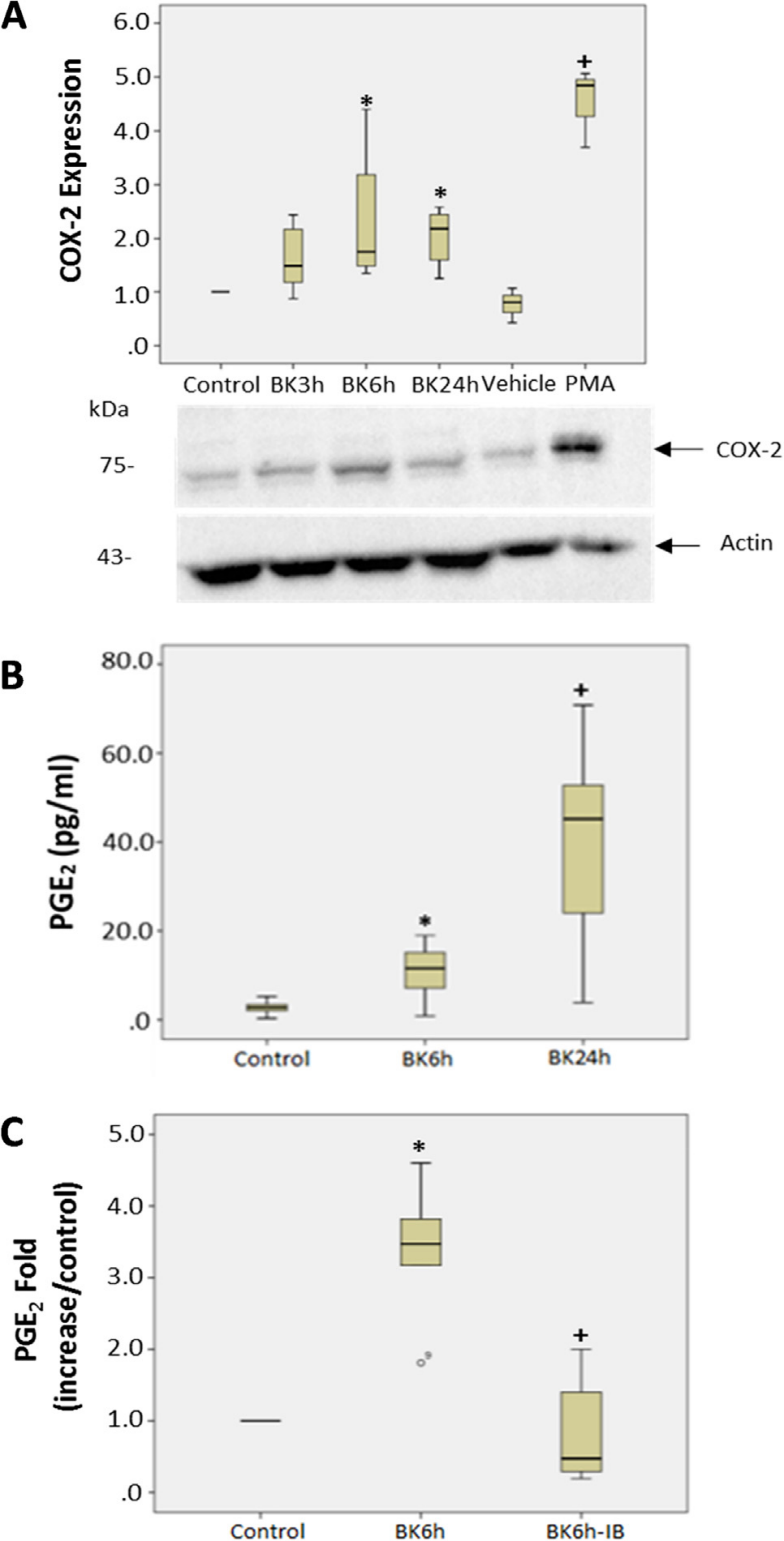
Effect of BK on inflammatory signals in podocytes **(a)** BK stimulated COX-2 protein expression in podocytes. Quiescent podocytes were stimulated with BK (10^−7^M) for 3, 6, and 24 h, and with PMA (10^−6^M) and DMSO was used as vehicle and did not exceed 0.1% (10^−6^ M). Bar graph represents the fold change in COX-2 protein levels (*p-value = 0.008 vs, control; +p-value = 0.05 vs DMSO) and correspond to the average of 3 or more separate experiments. **(b)** BK stimulated PGE_2_ release in podocytes. Quiescent podocytes were stimulated with BK (10^−7^ M) for 3, 6, and 24 h. Bar graph represents the fold change in measured PGE_2_ levels (*p < 0.001 BK 6 h vs Control, +p < 0.001 BK 24 h vs Control), and is the sum of 3 or more separate experiments. **(c)** Ibuprofen inhibits BK-stimulated PGE_2_ release in podocytes. Quiescent podocytes were stimulated with BK (10^−7^ M) for 6 h in the presence and absence of Ibuprofen (10^−6^ M). PGE_2_ levels were measured by EIA. Bar graph represents the fold change in measured PGE_2_ levels (*p < 0.016 BK 6 h vs Control, +p = 0.016 BK 6 h-IB vs BK 6 h) and is the average of at least 3 separate experiments.

The proteomic analysis data depicting the effects of BK on COX-2 upregulation by LC-MS/MS were validated by treating quiescent podocytes with BK (10^−7^M) for different time points (3, 6 and 24 h). The 24 h time point was studied to assess the temporal expression of COX-2 in response to BK and to determine the time at which the expression of COX-2 peaks in response to BK. Podocytes were also stimulated with phorbol-12-myristate-13-acetate (PMA), a phorbol ester as a positive control for COX-2 expression. The results indicate that the protein levels of COX-2 are higher at 6 and 24 h post BK stimulation compared to control unstimulated cells (Fig. 4A, p < 0.008). PMA, which is a positive control, also increased the protein levels of COX-2 compared to DMSO treated podocytes (p < 0.05, Fig. 4). We next assessed if the increase in COX-2 protein level in response to BK is correlated with an increase in COX activity by measuring the production of the downstream metabolite PGE_2._ PGE_2_ levels were significantly higher in podocytes at 6 and 24 h post BK treatment compared to unstimulated cells indicating that BK increased the formation of PGE_2_ consequent to the induction of COX-2 expression (p < 0.0001, Fig. 4B). To further evaluate the effect of BK on COX-2, we studied the effects of a COX inhibitor on the production of PGE_2_ levels in response to BK. Podocytes were stimulated with BK for 6 h in the presence and absence of the COX inhibitor Ibuprofen (10^−6^ M). The results shown in Fig. 4C, demonstrated that the increased production in PGE_2_ in response to BK was significantly attenuated in the presence of Ibuprofen (p < 0.015, Fig. 4C).

**Fig. 5:**
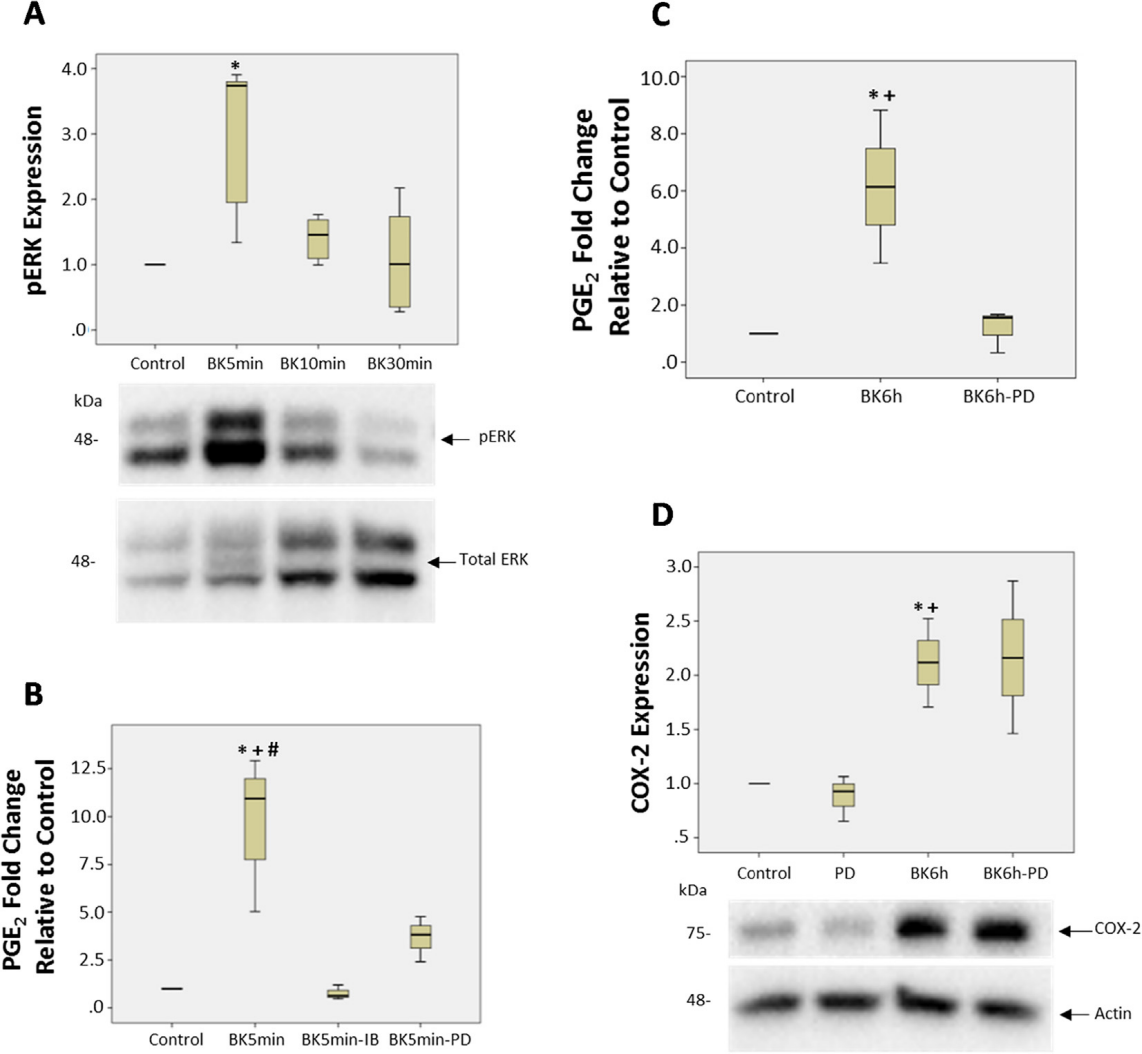
Role of extra cellular regulated kinase1/2 (ERK1/2) in BK stimulated expression of COX-2 and PGE2 production. **(a)** BK stimulated ERK1/2 phosphorylation (pERK 1/2) in podocytes. Quiescent podocytes were stimulated with BK (10^−7^ M) for 5, 10, 30 min. pERK and total ERK levels were assessed by western blot. Bar graph represents the fold change in pERK relative to total ERK protein levels and is the sum of 3 separate experiments (*p = 0.016). **(b)** Quiescent podocytes were stimulated with BK (10^−7^ M) for 5 min (5 min) in the presence and absence of PD98059 (25 μM) or Ibuprofen (10^−6^ M). Bar graph represents the fold change in measured PGE_2_ levels and is the sum of at least 3 separate experiments (*p-value = 0.004 BK 5 min vs Control; +p-value = 0.024 BK 5 min vs BK 5 min-IB; #p-value = 0.024 BK 5 min vs BK 5 min-PD). **(c)** Quiescent podocytes were stimulated with BK (10^−7^ M) for 6 h in the presence and absence of PD98059 (25 μM). Bar graph represents the fold change in measured PGE_2_ levels and is the sum of at least 3 separate experiments (*p < 0.03 BK 6 h vs Control, +p < 0.05 BK 6 h vs. BK 6 h-PD). **(d)** Quiescent podocytes were stimulated with BK (10^−7^ M) for 6 h in the presence and absence of PD98059 (25 μM). COX-2 and actin protein levels were measured by western blot. Bar graph represents the fold change in COX-2 proteins levels relative to β-actin protein levels and is the average of 3 separate experiments (*p-value = 0.035, BK 6 h vs. control; +p-value = 0.01 BK 6 h vs PD).

***Role of extracellular regulated kinase ½ (ERK1/2) on COX activity in response to BK***. The canonical pathway analysis generated by IPA of our proteomic data, suggested that the ERK1/2 pathway to be involved in the response of podocytes to BK treatment (Fig. 2b and 2c). To validate this finding, we first determined whether BK stimulates the activation of ERK1/2 in podocytes. Treatment of podocytes with BK (10^−7^M) for various times stimulated the phosphorylation of ERK1/2 compared to unstimulated cells, with a peak response at 5 min (p < 0.016, Fig. 5a). We next evaluated whether ERK1/2 plays a role in the production of PGE_2_ levels in response to BK treatment. PGE_2_ levels were measured in podocytes stimulated with BK (10^−7^M) for 5 min and for 6 h in the presence and absence of the MEK inhibitor PD98059 (25 μM). PD98059 is a selective and effective inhibitor of MEK 1 (MAPkinase kinase) an upstream activator of ERK1/2. PD98059 binds to MEK1 in its inactive state and hence stops its activation by c-Raf. Once inactivated, MEK1 will no longer be able to phosphorylate and active its downstream target ERK1/2. The results shown in Fig. 5B and 5C, demonstrate that the production of PGE_2_ levels in response to BK were significantly inhibited in the presence of the ERK1/2 inhibitor in the early time point (5 min, p < 0.024, Fig. 5B) as well as at 6 h (p < 0.05, Fig. 5C). We also evaluated the effects of Ibuprofen (10^−6^ M) on PGE_2_ production in response to BK stimulation for 5 min. The results demonstrate the PGE_2_ production in response to BK was significantly inhibited in the presence of the COX inhibitor (p < 0.024, Fig. 5B). Finally, we assessed whether the inhibition of the ERK1/2 pathway by PD98059 would influence the BK-induced COX-2 protein levels. The results shown in Fig. 5D, demonstrate that inhibition of ERK1/2 pathway did not significantly alter COX-2 protein levels in response to BK stimulation.

**Fig. 6:**
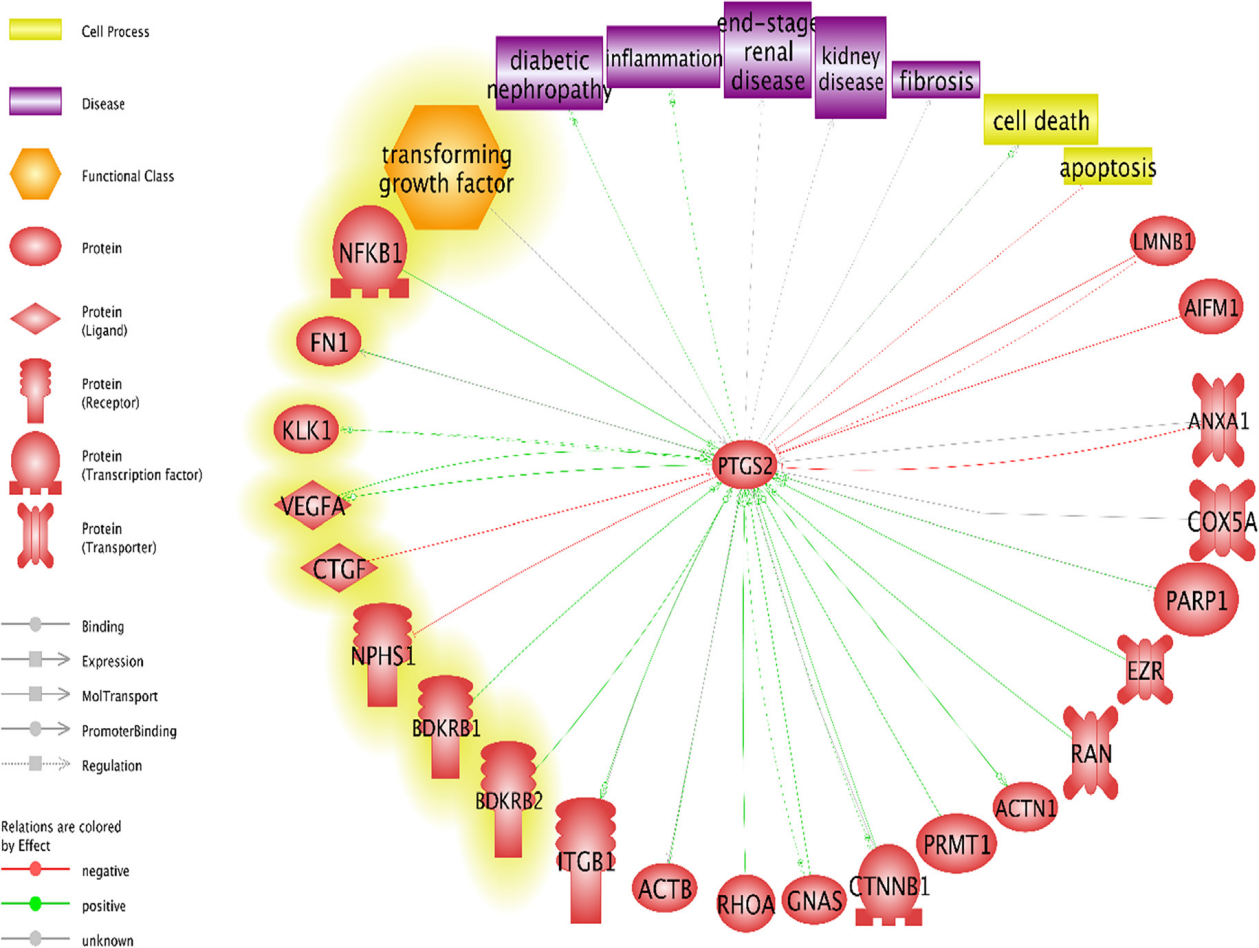
Pathway Studio analysis of the relationship for PTGS-2/COX-2 to the linked proteins from the 3 experiments according to the software, and to inputted proteins highlighted in yellow, cell processes in yellow boxes, and diseases related to cell death and diabetic nephropathy in purple boxes, with the software constructing extensive links between the different elements according to its knowledgebase (NFB1: Nuclear factor Kappa B subunit 1, FN1: Fibronectin 1, KLK1: Kallikrein 1, VEGFA: Vascular endothelial growth factor A, CTGF: Connective tissue growth factor, NPHS1: Nephrin, BDKRB1: Bradykinin receptor B1, BDKRB2: Bradykinin receptor B2). (For interpretation of the references to color in this figure legend, the reader is referred to the web version of this article.)

## Discussion

The advent of proteomic analysis has transformed the way we understand the hidden cellular processes and networks, and by extension, the different cellular phenomena and pathologies these networks might be interrelated to. Our global proteomic analysis of podocytes using shotgun proteomics has shed light on the different cellular processes occurring in response to BK treatment that are involved in podocyte cell injury. Some of the proteins of particular interest that have been identified from canonical pathways as well as those based on the suggested interactions with other proteins and relevant cellular processes as highlighted by our systems biology techniques included; inflammatory cytokines (IL-1) and chemokines, Calpain signaling, Granzyme signaling, Ephrin B signaling, RhoA, β-catenin, integrin-β1, apoptosis-inducing factor (AIFM-1), and PTGS-2 (COX-2). Calpain signaling is linked to modulation of increased production of proinflammatory mediators and activation of podocyte apoptosis [20]. Moreover, the increase in inflammatory cytokines and chemokines in response to BK is another facet through which BK contributes to glomerular and podocyte injury [21], [22]. Enhancement of Granzyme signaling by BK, a cluster of serine proteases generated by granules in the cytoplasma with cytotoxic T cells promote podocyte cell injury and apoptosis by enhancing the activity of caspases [23]. Furthermore, BK may influence podocyte function by enhancing the Ephrin B signaling, which interfaces with nephrin to sustain the architectural integrity and role of the slit diaphragm [24]. RhoA has generally been identified as a classic player in the cytoskeletal rearrangement pathway [25], which is of importance to us considering that podocyte cell death is usually accompanied by cytoskeletal rearrangements in the form of detachment from the glomerular basement membrane [26]. This result is consistent with another study showing a downregulation of RhoA in a podocyte injury model, and where the knock-down of RhoA by siRNA has shown increased podocyte apoptosis [27]. β-Catenin is another protein implicated in cytoskeletal rearrangement activities, as it interacts with the cytoplasmic regions of cadherin, serves the crucial role of regulating the adherent junctions, as well as participating as a key player in the Wnt signaling pathway [28]. Previous research also indicated a decrease in β-catenin in a DKD model, where both the protein levels and the mRNA levels of β-catenin were decreased in hyperglycemic conditions with the presence of advanced glycation end products (AGEs) [28], consistent with the decrease observed in our proteomics data. Integrin-β1 has also been associated with podocyte biology, where a study showed that mice with specific integrin-β1 deletions cannot complete postnatal renal development, and die of proteinuria [29], suggesting a possible role of the downregulation of integrin-β1 in the progression of DKD. Of interest, AIFM-1 is a protein that is recruited to the nucleus when apoptotic signals are initiated, where it will lead to DNA fragmentation [30]. However, no studies have been conducted on the activation of AIFM-1 in podocytes, especially that podocytes undergo apoptosis in DKD [26].

Our proteomic analysis is the first to show that BK induces the expression of COX-2 in podocytes. This finding was also confirmed by assessing the protein abundance of COX-2 levels in podocytes in response to BK stimulation. Upregulation of COX-2 has been implicated in DKD, for its specific inhibition in podocytes reduced proteinuria in a glomerular injury model [31]. In addition, COX-2 upregulation was also observed in glomerular podocytes in models of DKD and the targeted overexpression of COX-2 in glomerular podocytes was associated with worsening of nephropathy in mice with type 1 diabetes [32], [33]. Treatment with COX-2 inhibitors was shown to attenuate the albuminuria observed as a result of DKD [34], [35]. Not only did BK increase the protein levels of COX-2 but PGE_2_ synthesis. This was demonstrated by measuring the production of PGE_2_ metabolite, which is one of the products of arachidonic acid metabolism generated by COX activity. The production of PGE_2_ in response to BK stimulation was inhibited with COX inhibitors suggesting that BK can directly or indirectly modulate COX-2 activity in podocytes.

To gain insights into the mechanisms through which BK activates COX-2, we investigated the role of ERK1/2 in this process, as suggested by the pathway enrichment analysis of our proteomic profiling analysis. Our results indicated that ERK1/2 phosphorylation was enhanced in response to BK and the increased production of PGE_2_ in response to BK was attenuated in the presence of ERK1/2 inhibitors. This finding implicates the ERK pathway in the mechanisms through which BK modulates the generation of PGE_2_ levels in podocytes and suggests that COX-2 is downstream of ERK1/2.

It is interesting to point out here that the COX metabolite PGE_2_ is implicated in inducing podocyte apoptosis. In this regard PGE_2_ has multifaceted properties depending on the context and cell types. It has been shown that PGE_2_ is implicated in inducing apoptosis in various cell types including T and B lymphocytes, lung fibroblasts, and mesangial cells [36], [37], [38]. In podocytes, PGE_2_ induced apoptosis in a Stat3-dependent pathway since inhibition of Stat3 improved the PGE_2_- or Adriamycin-dependent induction of podocyte injury [39], [40]. In addition, the apoptotic effects of COX-2 expression and PGE_2_ receptor EP1- signaling were involved in a model of calcineurin induced podocyte apoptosis and injury [41]. This is in line with the systems biology analysis that predicted physiological and pathological processes underlying the change in protein profile of podocytes in response to BK as well as the pathways linking our observed proteins with other suggested proteins. The main processes suggested to be occurring were those related to cell death (apoptosis, necrosis, and cell death) and those related to cytoskeletal remodeling (microtubule dynamics and transport of molecules). While the microtubule dynamics was suggested to be upregulated and the “Transport of Molecules” suggested to be downregulated, cytoskeletal activity plays a role in podocyte function and death, where podocyte detachment goes hand-in-hand with podocyte loss and progression of kidney disease [42]. The majority of these pathways suggest a regulation of cytoskeletal elements of the podocytes, which is of particular importance given that podocyte death is associated with detachment from the globular basement membrane.

The data described herein support the current paradigm of thinking that activation of kallikrein leads to cleavage of kininogen to release the proinflammatory peptide BK [43], [44]. BK activates its constitutively expressed B_2_R in the kidney in an autocrine and or paracrine fashion to generate a plethora of cellular signals that impact renal function [11]. Inactivation of B_2_R significantly attenuated protein excretion in diabetic mice and improved the augmented nephropathy in uninephrectomized db/db mice, lending support to the detrimental function of B_2_R in DKD [45]**.** Furthermore, diabetic B_2_R knockout mice exhibited decreased AER and attenuated tubular and glomerular injury, thus implicating a harmful role for B_2_R in DKD [10]. However, other studies have shown that BK and its B_2_R play a beneficial role in DKD. B_2_R knockout mice crossed with the insulin Akita (Ins2^Akita^) mice to generate the double knockout (In2^Akita^/B_2_R^−/−^) exhibited augmented albuminuria related to Ins2^Akita^ mice alone [46]. Expression of B_2_R were shown to be attenuated in the glomeruli of STZ-diabetic rats and in podocytes cultured under hyperglycemic conditions. In addition, BK treatment attenuated the increased AER observed in diabetic rats and inhibited podocyte apoptosis [47]. The seeming changes in the function of B_2_R in DKD could be ascribed to variations in the DKD models studied, metabolic state and genetic background of animal models and essentials of the investigational approach and target variables evaluated.

It is important to point out here that some of the effects we observed in response to BK could conceivably be mediated via activation of B_1_-receptors expressed in podocytes. In this regard, it is feasible that once podocytes are exposed to BK, the peptidases that are expressed in podocytes can cleave BK to generate des-Arg^9^-BK, the B_1_-receptor ligand, which in turn will bind to its receptors in an autocrine manner to transduce its signal. This possibility will be verified in future studies.

## Conclusion

The findings of the present manuscript lends further support to our hypothesis that engagement of B_2_ receptors contribute to DKD development and establish that BK enhances the expression of COX-2 and stimulates that production of PGE_2_, factors that are associated with promotion of albuminuria and podocyte apoptosis (Fig.6). The current findings offer new understandings into mechanisms through which BK signaling processes modulate the pathogenesis of DKD and identify new targets for therapy.

## Compliance with Ethical Requirements

This article does not contain any studies with human or animal subjects

## Declaration of Competing Interest

The authors declare that they have no known competing financial interests or personal relationships that could have appeared to influence the work reported in this paper.
